# Impact of the Las Vegas Mass Shooting Event on the Graduate Medical Education Mission: Can There Be Growth from Tragedy?

**DOI:** 10.5811/westjem.2022.9.56221

**Published:** 2022-12-09

**Authors:** Gregory T. Guldner, Suzanne M. Roozendaal, Ross P. Berkeley, Michael P. Allswede, Kristina H. Domanski, Obadha M. Sairafe, Dylan F. Davey, Hoda Abou-Ziab, Jason T. Siegel

**Affiliations:** * University of California Riverside School of Medicine, Department of Emergency Medicine, Riverside, California; † HCA Healthcare GME, Brentwood, Tennessee; ‡ Sunrise Health GME Consortium, Department of Emergency Medicine, Las Vegas, Nevada; § Kirk Kerkorian School of Medicine at UNLV, Department of Emergency Medicine, Las Vegas, Nevada; ¶ Claremont Graduate University, Division of Behavioral & Organizational Sciences, Claremont, California; || Sunrise Health GME Consortium, Department of Surgery, Las Vegas, Nevada; # University of California Riverside School of Medicine, Riverside Community Hospital, Department of Health Sciences, Riverside, California

## Abstract

**Introduction:**

Our aim was to determine the psychological and educational impact of the 2017 Las Vegas mass shooting on the graduate medical education (GME) mission within two cohorts of resident physicians and attending faculty at two nearby academic trauma centers.

**Methods:**

A cross-sectional survey assessed 55 resident physicians and attending faculty involved in the acute care of the patients from the mass shooting. We measured the psychological impact of the event, post-traumatic growth, team cohesion, social support, and known risk factors for post-traumatic stress disorder (PTSD). Additionally, we assessed the impact of the event on GME-specific tasks.

**Results:**

Attending faculty and physicians in training in GME residencies evaluated over 300 penetrating trauma patients in less than 24 hours, and approximately 1 in 3 physicians had a patient die under their care. Despite this potential for psychological trauma, the majority of clinicians reported minimal distress and minimal impact on GME activities. However, 1 in 10 physicians screened positive for possible PTSD. Paradoxically, the minority of physicians who sought psychological counseling after the event (20%) were not those who reported the highest levels of distress. Residents generally assessed the event as having an overall negative impact on their educational goals, while attendings reported a positive impact. Psychological impact correlated inversely with social support and the amount of prior education relating to mass casualty incidents (MCI) but correlated directly with the degree of stress prior to the event.

**Conclusion:**

Despite the substantial level of exposure, most resident physicians did not report significant psychological trauma or an impact on their GME mission. Some reported post-traumatic growth. However, a minority reported a significant negative impact; institutions should consider broad screening efforts to detect and assist these individuals after a MCI. Social support, stress reduction, and education on MCIs may buffer the effects of future psychologically traumatic events on physicians in training.

## INTRODUCTION

Teaching hospitals serve a dual role, providing for both graduate medical education (GME) and patient care. This necessarily intertwines teaching and learning activities with unpredictable and traumatic patient care events such as mass casualty incidents (MCI). Mass shootings, which occur in the United States at a rate of approximately one every 12.5 days,^1^ represent a subset of MCIs with a potential to inflict profound psychological distress on physicians. Exposure to such events may lead to disruption of personal and professional activities and lead to acute stress disorders, sleep disturbances, anxiety, depression, complicated grief, and psychological distress.^2^

One such event occurred on October 1, 2017, when an individual armed with multiple weapons opened fire on spectators at the Route 91 Harvest Music Festival in Las Vegas, Nevada. This event was the deadliest mass shooting in US history^3^: 58 people died at the scene or soon thereafter, with two additional deaths from subsequent complications of injuries. Over 400 additional patients sustained penetrating injuries from gunshots and/or shrapnel, with hundreds of other injuries sustained in the subsequent panic of the crowd.^4^ The overwhelming volume and acuity of critically injured patients exceeded the capacity of emergency medical services at the concert venue, and large numbers of patients were transported to hospitals by private vehicles. Two nearby trauma centers, Sunrise Hospital and Medical Center (SHMC) and University Medical Center of Southern Nevada (UMC), both academic training facilities, received most of the injured patients and cared for hundreds of gunshot patients over the span of a few hours. The attending and resident physicians from these centers were exposed to an extraordinary number of emotionally intense traumatic injuries, far beyond the typical experience of clinicians.

Psychologically traumatic exposures such as this can lead to significant post-traumatic stress for some healthcare workers.^5^ The emergency department (ED) setting, where attending and resident physicians stabilize victims of trauma, has historically reported high rates of post-traumatic stress.^6^ Rates of post-traumatic stress disorder (PTSD) range from 2.2–24%, depending on definition and measurement technique.^5^ Affected physicians may show typical signs of post-traumatic stress including involuntary upsetting memories, flashbacks, involuntary responses to external cues, and physical symptoms such as palpitations upon exposure to a reminder of the event.^7^ However, most physicians exposed to psychologically traumatic events do not develop PTSD. Thus, systemic or personal factors, beyond mere exposure, contribute to the psychological impact of a traumatic event on an individual physician.^8^

**Population Health Research Capsule**

What do we already know about this issue?

*Caring for patients from mass shootings and catastrophes can impact clinicians’ personal and professional lives, with a minority developing symptoms of post-traumatic stress disorder*.

What was the research question?

*How did the Las Vegas mass shooting impact the academic activities of the residents and faculty involved?*

What was the major finding of the study?

*The Impact on academics was negative for residents, positive for attendings (2.5 vs 4.9 on 1 to 7 scale; P < 0.01)*.

How does this improve population health?

*Academic trauma centers can better understand the complex impact of mass shooting events on their physicians, potentially resulting in improved care for their community*.

The best researched systemic factor demonstrating a consistent inverse relationship with the risk of development of PTSD is the degree of social support.^9^ Prior training in stressful events, and methods of coping with the psychological aftermath, have also correlated with a protective effect against the development of PTSD.^10^,^11^ Individual factors linked to the development of PTSD include age (older individuals are at higher risk), the amount of professional training and education (more years are protective), and the presence of pre-existing mental health disorders (those impacted are at increased risk).^5^ While traumatic events may cause substantial negative impact, for some individuals trauma may lead to growth. Post-traumatic growth is a well-researched process in which an individual exposed to a psychologically traumatic event undergoes productive personal development leading to higher functioning.^12^ Post-traumatic growth occurs through changes in one or more of several psychological domains: appreciation of life, relationship with others, new possibilities or purpose in life, personal strength, or spiritual change.^13^

While several studies have looked at the impact of stressors such as pandemics and MCIs on physicians’ personal lives, and to a lesser extent their professional lives, there is a paucity of evidence on the impact of traumatic events specifically on the GME mission. The lack of understanding of this potential impact is especially concerning given that academic hospitals, which are frequently larger urban trauma centers, often provide a disproportionate amount of patient care during these MCIs. Thus, in our study we sought to evaluate the self-reported impact of the deadliest mass shooting event in US history on peri-traumatic stress, post-traumatic growth, and GME-related activities among residents and attending faculty at two teaching hospitals.

## METHODS

### Study Design and Subjects

Approximately six months following the shooting, we performed a post-exposure, cross-sectional survey involving resident and attending physicians who were present during the event at the two teaching hospitals impacted: SHMC is a Level II trauma center that treated over 200 mass casualties that evening, and UMC is a Level I trauma center that treated 104 patients. The timing of the survey represented the earliest point that the researchers were able to develop a protocol and obtain institutional review board (IRB) approval at both institutions following the event. No contemporaneously recorded logs of physicians present during the event exist due to the chaos that evening and the addition of unscheduled clinicians from multiple disciplines who arrived on scene spontaneously. Thus, we obtained a list from both institutions of all credentialed physicians who had potentially assisted during the mass shooting event. All physicians were contacted by email with an introduction to the study, a link to the survey, and a request for participants who were involved in caring for the patients from this event either the evening of October 1, 2017, or the morning after. Those who confirmed involvement were included in the study. The study was approved by the IRBs of both SHMC and UMC.

### Assessment Tools

To determine the range of exposure to potentially psychologically traumatic events participants were asked a series of “yes” or “no” questions regarding their overall involvement in the events following the shooting. While many participants assumed direct patient care roles some may have provided non-clinical activities such as assisting with supplies, providing information, or offering psycho-social support. These questions included the following:

- *Did you personally provide direct care to a shooting victim?*
- *Did you personally have a patient from the Las Vegas shooting, who you were treating, die during your care?*
- *Did you personally have to inform relatives or loved ones of a patient’s death?*
- *Did you personally witness images resulting from violence that were out of the ordinary for you as a physician?*
- *Did you feel personally at risk of injury or death during the event?*

The survey included four previously psychometrically validated scales as outcome measures. The Impact of Events Scale – Revised (IES-R) is a 22-item self-report of the degree of subjective distress following a traumatic event. Respondents assess the degree to which they experience each item on a five-point scale ranging from “not at all” to “very much.”^14^ Post-traumatic growth was assessed with the Post Traumatic Growth Inventory - Short Form (PTGI), a 10-item scale with ranges between zero (“did not experience this”) and five (“experienced to a very great degree”).^15^ This scale captures the degree of positive changes in each of the five domains of growth that may occur following a traumatic event.

We assessed the impact of environmental factors using two scales: the Multidimensional Scale of Perceived Social Support (MSPSS) and four items measuring team cohesion from the Team Development Measure. The MSPSS is a 12-item scale with ranges between 1 (“very strongly disagree”) and 7 (“very strongly agree”) that captures perceived social support from family, friends, and significant others.^16^ The team cohesion factor (TCF) consists of a four-item scale that measures the degree to which the respondent feels the team they were on was united and that they personally contributed to the overall mission of the team, using a five-point Likert scale from “strongly agree” to “strongly disagree.”^17^

In addition, several questions were asked to assess the perceived personal impact of the event, scored on a seven-point Likert scale. These questions were developed by a review of the literature, item development, and then content validation by group discussion among authors with expertise in clinical psychology. The question structure was developed by one author who is an academic psychologist with expertise in survey design methodology. These questions included the following:

- *How frequently have you found yourself avoiding a particular type of patient? For example, avoiding treating patients with penetrating trauma*. [Anchors of “Never” to “All the Time”]
- *How frequently have you found yourself having difficulty taking care of a particular type of patient? For example, having strong emotions while treating patients with penetrating trauma*. [Anchors of “Never” to “All the Time”]
- *In general, how would you say the Las Vegas shooting experience impacted your academic clinical practice? (Ability to teach, model, and perform in GME)?* [Anchors of “Strong Negative Impact” to “Strong Positive Impact”]
- *I have considered changing my specialty because of the event*. [Anchored “Strongly agree” to “Strongly disagree”]
- *I have considered leaving the field of medicine because of the event*. [Anchored “Strongly agree” to “Strongly disagree”]

Other known risk factors for peri-traumatic stress were assessed by the following questions:

- *Prior to the shooting, did you ever seek treatment for any of the following conditions? Anxiety, depression, PTSD, obsessive/compulsive disorder, personality disorder, Any other mental health condition*. [Coded as Yes/No]
- *Other than the Las Vegas shooting have you previously had an exposure to an event you considered to be psychologically traumatic to you?* [Coded Yes/No]
- *In the seven days leading up to the shooting, how stressed would you say you were?* [Coded on a seven-point Likert scale from “Not at all” to “Extremely”]
- *Prior to the shooting, approximately how much prior formal training had you had regarding mass casualty events?* [Coded as “none,” “1–2 hours,” “2–3 hours,” “3–4 hours,” or “more than 4 hours”]
- *Prior to the shooting, approximately how much formal training had you completed regarding the psychological impact of critical incidents such as the shooting?* [Coded as “none,” “1–2 hours,” “2–3 hours,” “3–4 hours,” or “more than 4 hours”]

Participant age was not included in the survey due to concerns about maintaining anonymity.

We determined types of GME activities common to residents and attendings by a review of the Accreditation Council for Graduate Medical Education Program Requirements and discussion between authors. Participants were asked, *“What impact, if any, has the event had on your ability to complete these education-related tasks?”* Responses for each type of activity fell on a nine-item Likert scale ranging from “Much Easier Now” to “Much Harder Now,” with the mid-range labeled as “No impact.” Table 1 shows the GME activity-related questions for attendings and residents.

### Statistical Analysis

We present the survey results with descriptive statistics (mean, standard deviation). Univariate associations between continuous variables were determined by Pearson product moment correlations. The association between binary “yes/no” questions and the IES-R and PTGI were measured by point biserial correlation. We measured the associations between both the IES-R and the PTGI with questions with ordinal answer sets (eg, “Less than 1 hour,” “1–2 hours,” or “2–3 hours”) with Spearman’s rho. We used a two-tailed Student’s *t*-test for comparisons between residents and attending physicians on continuous variables that were normally distributed according to the Shapiro-Francia test for normality. Non-normally distributed variables were compared with the Mann-Whitney U test. To determine whether the event differentially impacted certain types of GME-related activities, we conducted two separate within-subjects ANOVAs for attendings and residents. There was minimal missing data, but when present in any given statistical analysis it was handled by listwise deletion. We calculated statistics with STATA version 15 (StataCorp., College Station, TX).

## RESULTS

### Description of Participants

A total of 320 physicians were contacted by email. Of these, 55 (17%) confirmed their involvement in the event and completed the survey: 38 attending faculty and 17 residents. We cannot determine the response rate as a function of all physicians who actually participated in the care of patients during the shooting (as opposed to all physicians credentialed at both hospitals) because no accurate record exists from the event itself. Of the attending physicians 15 identified as emergency medicine (EM), eight as general surgery, four as anesthesiology, three as surgical subspecialties, and two as radiology, while six did not identify their role. Of the residents, there were 11 general surgery, four EM, and one family medicine resident, and one who did not identify their role.

### Psychological Impact and Comparisons by GME Role

Table 2 shows the degree of exposure to psychologically traumatic events reported by study participants, and Table 3 shows the summary results of our outcome variables. The results of the IES-R (psychological impact) and PTGI (post-traumatic growth) did not differ by group when comparing those who endorsed specific exposures (directly provided care, had a patient die in their care, participated in death notification) to those who did not. Six of 15 residents (40%) and 19 of 36 attendings (53%) endorsed a prior psychologically traumatizing experience. (Two in each group did not answer.) This ratio does not differ by role (chi square = 0.27, *P* = .60). Four of 15 residents (27%) and six of 36 attendings (17%) endorsed a prior mental health condition. (Two in each group did not answer.) This ratio does not differ by role (chi square = 0.69, *P* =.41). Comparing those participants who endorsed prior mental health conditions to those who did not we found no statistically significant differences in social support (MSPSS), psychological impact (IES-R), team cohesion, PTGI or the global impact question. We found a similar lack of significant differences when comparing those participants who endorsed a prior psychologically traumatic experience to those who did not. The large majority of both attendings (89%) and residents (82%) reported no subsequent difficulties involving either avoiding certain types of patients (eg, trauma) or with distress associated with seeing certain types of patients after the event. Few participants reported they would consider either changing specialties (4%) or leaving medicine (7%) specifically as a result of exposure to this event.

The mean and standard deviation of the standardized scales and the degree of stress prior to the event are shown in Table 4. All scales were non-normally distributed. Residents and attendings did not differ on the IES-R, PTGI, TCF, or the degree of stress perceived prior to the event. Residents reported slightly higher social support. Four of the 38 attending physicians (11%) and two of the 17 residents (12%) scored above the standard cutoff of 24 to signal concern for PTSD. Two of the four attending physicians scored above 33, the standard cutoff for “probable” PTSD.^18^ Participants who screened positive for PTSD came from EM, anesthesiology, and one additional specialty in the “other” category. Only three of the 17 residents (18%) and seven of the 34 attendings (21%) undertook specific formal efforts to mitigate the psychological impact of the event. Notably, of the six participants scoring above the cutoff on the IES-R signifying more distress, only one also noted a formal effort to mitigate the impact.

The single-item global assessment of impact on GME was normally distributed and differed significantly between residents and attendings by two-tailed *t*-test (*t* = 7.03, df = 50, *P* < 0.01). Residents reported an overall negative impact with a mean score of 2.53 on the seven-point scale (SD = 1.33), and attendings reported an overall positive impact with a mean of 4.83 (SD = 0.98). A score of 4 on the scale is anchored as “no effect.”

### Univariate Associations with Peri-Traumatic Stress and Post-traumatic Growth

Table 5 demonstrates the correlation matrix for the standardized scales and the degree of stress prior to the event for all participants (residents and attending physicians combined). Team cohesion correlated positively with the degree of social support. However, this relationship held only among the residents (r = 0.50, *P* < 0.05), not among the attendings (r = 0.10, *P* = 0.55).

### Training in Mass Casualty Incidents and Psychological Trauma

Of the residents, 11 (65%) reported some prior training on MCIs, with six (35%) reporting some training on the psychological impact of MCIs on clinicians. Attending physicians more frequently reported some exposure to MCI training (31 of 38 [82%]) and its psychological impact (22 of 38 [58%]). Combining both groups, the average amount of training on MCIs was 2–3 hours with an average of 1–2 hours on the psychological impact. Among residents, IES-R correlated inversely with the amount of MCI training (r = −0.67, *P* < 0.01), and with attendings, IES-R correlated inversely with the amount of training on the psychological effects of MCI (r = −0.39, *P* = 0.02). None of the other ordinal or binomial variables (attended to a patient, witnessed a patient’s death, performed death notification, or prior diagnosis of mental health condition) correlated at a statistically significant level with IES-R for residents or attendings. None of the ordinal or binomial variables correlated at a statistically significant level with the PTGI for either residents or attendings.

### Univariate Associations with Graduate Medical Education-specific Tasks

Overall, a majority of both residents and attendings reported that the mass shooting had little effect on GME-specific activities. Attendings in particular reported minimal impact, with 94% of responses relating to GME-specific activities reported as “no impact,” 2% indicating “negative impact,” and 4% indicating “positive impact.” Among the residents, 71% of the GME-specific activities were rated as “no impact,” 18% as “negative impact,” and 11% as “positive impact.” Analysis of the distribution of responses revealed that there were outlier participants responsible for the majority of non-neutral responses. Two of the 17 residents (12%) scored, on average, more than two standard deviations above the mean (reflecting that GME activities were much harder). Seven of the 34 attending physicians (21%) who answered all items scored, on average, two standard deviations below the mean (reflecting that GME activities were easier).

The relationship between the overall impression of the impact of the event on GME tasks and post-traumatic growth differed between residents and attendings. Among attendings, the more positive impact they felt the event had on GME tasks, the more post-traumatic growth they reported (r = 0.33, *P* = 0.05). However, with residents this relationship was reversed, although not statistically significant, likely due to the smaller sample size (r = −0.31, *P* = 0.23).

## DISCUSSION

We sought to determine the potential psychological and educational impact of the worst mass shooting event in US history on members of the GME community who cared for the patients. Consistent with prior literature, most participants, both attendings and residents, reported relatively low levels of post-traumatic stress symptoms five to six months after the event. The vast majority of participants did not intend to either leave medicine or change specialty as a result of this specific exposure to a MCI. Roughly 1 in 10 participants reported symptoms severe enough to be considered PTSD. A previous prevalence screening study of 190 physicians at trauma centers in Texas found a similar rate, with 13% reporting they had previously sought treatment for PTSD-type symptoms.^6^ In the Texas study, 16% of ED attendings, 29% of EM residents, and 22% of surgery residents screened at risk for PTSD. Surprisingly, not one of the 15 trauma surgeons screened positive. This is consistent with our current study, which also showed no surgery attending or resident endorsing a level of psychological impact that would suggest PTSD. However, other studies of trauma surgeons have demonstrated a 15% rate of probable PTSD.^19^ Thus, it remains unclear whether surgery selects for or develops individuals with a lower risk of PTSD overall or whether social response or selection bias accounts for the lack of surgeons endorsing mental health symptoms in some studies. Importantly, almost all studies used short screening surveys to screen for PTSD, which may overestimate the true rates of clinical PTSD. A study using a survey comprised of the full *Diagnostic and Statistical Manual of Mental Disorders*, 5^th^ ed, criteria found a PTSD rate of 2.2% in physicians.^20^

This suggests that efforts to mitigate the impact of psychologically traumatic events in the GME community should begin with screening to detect those more likely to benefit from additional interventions, rather than comprehensive trauma intervention programs designed for all involved. Most individuals exposed to psychological trauma do not develop PTSD,^21^ and early interventions such as mandatory debriefing sessions for all clinicians have not demonstrated efficacy.^22^

Despite the profound potential of the Las Vegas mass shooting to create psychological trauma, we found that few physicians chose to mitigate the impact with help-seeking behaviors. Paradoxically, the few participants who did seek help were not those with the highest reported distress. This inverse relationship between help-seeking and degree of distress is also seen in the depression literature, which has shown that the most distressed individuals are often the least likely to seek help.^23^ This pattern has serious implications regarding the typical institutional practice of suggesting to physicians, “If you need help, ask for help.” Those who most need help often will not ask.

Regarding GME-specific tasks, the majority of both attendings and residents reported minimal to no impact from the event. Overall, the impact on educational activities was independent of the psychological impact of the event, as evidenced by the near-zero correlation between the IES-R and the global assessment of educational impact. However, residents differed from attendings in their assessment of the educational impact on GME activities. Residents reported the event as negatively impacting their GME experience, while attendings presumably reframed the event as one in which growth occurred. This bias toward growth was seen despite the fact that attendings, far more than residents, correctly recognized the MCI as “outside the normal experience for a physician.” Residents may have perceived the MCI as creating a substantial increase in workload. This may have created a work and learning environment perceived as too heavily focused on clinical service vs education. Additionally, residents faced with increased work demands may not have recognized the potential educational impact of the MCI.

Despite reporting similar profiles in overall psychological impact, post-traumatic growth, team cohesion, and perceived stress, attending physicians perceived the impact of this traumatic event on the didactic environment differently than the resident physicians. Some possible explanations may include differences in age, psychological resources, sense of purpose, autonomy, confidence in patient care, or commitment to an organization.

The retrospective assessment of the impact of the event may be a function of the demands placed on the individual and the resources they employ to meet those demands. For example, since residents do not possess a complete skillset and work under supervision, it is possible they may have experienced a greater sense of helplessness, which has been linked to the development of peri-traumatic distress.^24^ It is also possible that an MCI may be perceived by certain experienced physicians as an opportunity to demonstrate competency, while for residents such an event may potentially expose weaknesses or knowledge gaps related to their level of training. Although residents did not report greater psychological distress, they did report a more negative perceived impact on their education. Residents’ primary developmental goal is professional growth toward independent practice, while attendings have achieved this milestone and are focused on various other objectives. The impact of an MCI appears to disrupt educational goals variably, more frequently for physicians in training, and only for a minority of residents.

Consistent with prior literature, we found that social support was inversely associated with distress. Social support plays a substantial role in overall well-being, as it mitigates depression, encourages work engagement, and buffers stressors in the environment.^25^ Deliberate institutional efforts to develop and sustain high levels of collegiality and perceived social support create positive work environments. This likely mitigates the psychological impact of catastrophic events such as the Las Vegas shooting on the healthcare team. Similarly, the association between perceived baseline stress prior to the event and subsequent psychological impact^26^ provides a target for institutions hoping to mitigate the impact of a similar event. Broad efforts to improve the workplace environment and lessen perceived stress on the GME community should be supported for many reasons. Our study demonstrates yet another domain in which the high levels of baseline stress can negatively impact GME physicians.

Prior studies have shown a relationship between the risk of PTSD and both a sense of helplessness and the degree of prior training in MCIs.^5^,^27^,^28^ Consistent with this finding we found an inverse relationship between prior training in MCIs, including training on their psychological impact, and the impact of a traumatic exposure on GME physicians. Institutions should prioritize training in MCIs and the psychological impact of these events as a strategy for mitigating clinician distress. These training events do not require inordinate time commitments. In our study incremental differences of 1–2 hours predicted less psychological distress.

For both cohorts, the degree of psychological impact positively correlated with post-traumatic growth: a relationship noted in prior research in general^29^ and specifically among emergency physicians.^30^ Post-traumatic growth arises out of the psychological struggle to integrate traumatic events with one’s prior understanding of the world. Further research is needed to explore the relationship between psychological trauma and growth in hopes of promoting positive individual development, rather than maladaptive behaviors, after exposure to trauma.

## LIMITATIONS

Our research has several limitations. The total number of physicians who actually participated in the care of patients during the Las Vegas MCI is unknown, and thus our survey response rate is unknown. The degree of individual distress may have impacted physicians’ willingness to participate in the survey, thereby biasing our study population to reflect a less generalizable cohort. Given that participants self-selected to complete the survey our cohort may suffer from selection bias, as the overall population of physicians who experienced the MCI may differ from those who agreed to participate. Similarly, given the anonymity of the study and the contemporaneous chaos of the event, we cannot confirm that all participants were actually involved in the event other than through their endorsement of being eligible for the study, nor can we determine the extent or nature of the experience of individual participants. While some participants were directly involved in patient care others may have been involved in providing ancillary services such as transportation, logistics assistance, or psycho-social support.

The survey was distributed five to six months following the mass shooting; therefore, participant responses reflect their understanding of the event after contemplation. A follow-up survey to assess trends and possible longer term impact of the event is under development. Typically, disasters create predictable psychological phases of various durations.^31^ Initially, the heroic and honeymoon phases last weeks to months and create a sense of social support and hope. The disillusionment phase follows when the realities of the impact of the disaster may be unopposed by the more positive support from the earlier phases. This may last between 3–36 months followed by the final restorative phase. Thus, the timing of our survey likely corresponds with the disillusionment phase; surveys conducted in earlier or later phases may have yielded different results.

Age, which is related to psychological distress and thus can be a confounder, was not assessed to avoid identification of any specific individual’s responses. However, the analysis by group (resident vs attending) serves as an imperfect proxy assessment of this variable. Due to the small sample size we did not attempt to compare various specialties to one another in their response to the event. Some specialties, such as the two radiologists who completed the survey, may have had a different level of exposure to trauma than other specialties. However, unlike in routine care, some radiologists came to the bedside during the event to interpret radiographs on portable imaging machines, exposing them to unusual scenes of violence.

Some of the measures, such as the TCF, asked participants about the level of teamwork at the time of the event which could have resulted in recall bias, as residents with greater or lesser overall impact may have recalled their team cohesion differently. The unpredictability of mass shootings creates significant barriers to any prospective research on the impact of psychological trauma on the GME mission.

Although we asked participants whether they sought psychological assistance following the event, we did not inquire as to the specific type of intervention obtained. Some evidence suggests a differential impact on post-traumatic symptoms depending on the type of psychological approach used; and we could not determine what approaches were employed in our sample population.

Future research on psychological trauma within the GME population may help better characterize the factors that determine the likelihood of an individual developing post-traumatic growth or PTSD symptoms. An examination of why some groups retrospectively view trauma with growth while others view it as entirely negative could yield valuable insights to assist future development of pre- and peri-event interventions.

## CONCLUSION

This study of 55 attending and resident physicians involved in the aftermath of the tragic events of the Las Vegas mass shooting found that, months after the event, most physicians reported low levels of PTSD symptoms and minimal impact on GME-specific activities. However, approximately 10% of both resident and attending physicians screened positive for possible PTSD. Attendings and residents differed in their overall global assessment of the impact of the event on their educational mission, with some attendings viewing it as resulting in growth while residents generally perceived it as either neutral or negative.

## Figures and Tables

**Table 1:** Graduate medical education (GME)-related survey questions by GME role.

Prompt: What impact if any has the event had on your ability to complete these GME-related tasks?
Attending Physician Reading or studying CME articles or other material relevant to training residents Participation in teaching rounds, educational half-days, noon conferences, or didactics Completing the required residency administrative tasks such as resident evaluations and time sheets Providing teaching during resident presentation of patients Performing procedures such as operating, intubating, chest tubes, etc. Communicating with patients and families Working on research projects or academic scholarly activities Recalling specific information when you need it (memory) Providing day-to-day feedback and guidance to residents
Resident Physician Reading or studying the material you need to know Participation in teaching rounds, educational half-days, noon conferences, or didactics Completing the required residency administrative tasks such as procedure logs, evaluations, and case logs Presenting patients to an attending, fellow, or senior resident Performing procedures such as operating, intubating, chest tubes, etc. Communicating with patients and their families Teaching medical students or other learners Recalling specific information when you need it (memory) Working on research projects or academic scholarly activities

*GME*, graduate medical education; *CME*, continuing medical education.

**Table 2:** Proportion of respondents with exposure to potentially traumatizing experiences.

	Residents N = 17	Attendings N = 38
Provided direct care for a shooting victim.	14 (82%)	30 (79%)
Patient died in the care of the participant.	7 (41%)	12 (32%)
Participant informed a relative or loved one about a death.	2 (12%)	2 (5%)
Personally witnessed images resulting from violence that were out of the ordinary for them as a physician.	5 (29%)	19 (50%)
Did you personally feel at risk of injury or death?	0 (0%)	0 (0%)

**Table 3:** Summary outcomes of measured variables.

Construct	Instrument	Outcome
Psychological impact/risk for PTSD	IES-R	4 of 38 attendings and 2 of 17 residents screened positive for possible PTSD. Of these, 2 attendings and 0 residents screened positive for probable PTSD.
Post-traumatic growth	PTGI	0 of 17 residents and 4 of 38 attendings scored at or above moderate post-traumatic growth cutoffs.
Perceived social support	MSPSS	Residents report slightly higher perceived social support than attendings.*
Team cohesion	TCF	Residents and attendings reported similar team cohesion.
Perceived stress prior to the event	Stress	Residents and attendings reported similar perceived stress prior to the event.
Prior mental health condition	Yes/no questions	6 of 26 attendings and 4 of 15 residents endorsed prior mental health conditions.
Prior psychologically traumatic event	Yes/no question	19 of 36 attendings and 6 of 15 residents endorsed a prior psychologically traumatic event.
Prior MCI training	Ordinal options	The modal response for both residents and attendings was >4 hours.
Prior training on psychological impact of MCIs	Ordinal options	The modal response for both residents and attendings was “none.” However, 6 of the 38 attendings and 4 of 17 residents reported > 4 hours.
Impact of event on core GME tasks	Ordinal options	Attendings: 94% “no impact,” 2% “negative impact,” and 4% “positive impact.” Residents: 71% “no impact,” 18% “negative impact,” and 11% “positive impact.”
Overall impact on GME	Likert Scale	Residents reported the event had a negative overall impact and attendings reported the event had a slightly positive overall impact.*

Unless noted, rates do not differ statistically between residents and attendings.

* P<0.05. Cutoff for IES-R “possible” PTSD is a score > 23 and “probable” PTSD is a score > 32.

Cutoff for PTGI for “moderate” post-traumatic growth is >29.

*IES-R*, Impact of Event Scales – Revised; *PTGI*, Post Traumatic Growth Inventory; *PTSD*, post-traumatic stress disorder; *MCI*, mass casualty incident; *GME*, graduate medical education.

**Table 4:** Outcomes of measured variables by graduate medical education role.

Construct	Instrument	Attending M (SD)	Resident M (SD)	Mann-Whitney U Test			

				U	Sample size**		*P*

					Att	Res	
Psychological impact/post-traumatic stress	IES-R	10.0 (12.5)	12.1 (17.1)	312.5	38	17	0.61
Post-traumatic growth	PTGI	12.5 (9.5)	10.8 (11.9)	306.5	38	17	0.54
Perceived social support	MSPSS	59.2 (17.7)	62.8 (17.0)	220.5	37	17	0.04*
Team cohesion	TCF	18.8 (2.5)	17.1 (4.0)	241.0	36	17	0.13
Perceived stress prior to event	Stress	3.2 (1.6)	3.5 (1.8)	284.0	38	17	0.31

* P < 0.05.

** Sample size varied due to missing data,

Cutoff for IES-R “possible” PTSD is a score > 23 and “probable” PTSD is a score > 32. Cutoff for PTGI for “moderate” post-traumatic growth is > 29.

Stress: Degree of perceived stress prior to the event, scored from 1 = “not at all” to 7 = “extremely.”

*M*, median; *IES-R*, Impact of Events Scale – Revised; *PTGI*, Post Traumatic Growth Inventory; *MSPSS*, Multidimensional Scale of Perceived Social Support; *TCF*, Team Cohesion Factor; *Att*, attending physician; Res, resident.

**Table 5:** Pearson correlation matrix for scales, stress, and general impression of event on graduate medical education.

	IES-R	MSPSS	PTGI	TCF	Stress	General impression
IES-R	1.00	−0.28*	0.50*	0.11	0.28*	0.01
MSPSS		1.00	0.20	0.27*	0.06	−0.12
PTGI			1.00	−0.05	0.13	0.03
TCF				1.00	−0.17	−0.23
Stress					1.00	−0.03

* P<0.05 by Pearson product moment correlation. N=55.

Stress: Degree of perceived stress prior to the event, scored from 1 = ”not at all” to 7 = “extremely.” General Impression: Scored 1 = “Strongly Negative” to 7 “Strongly Positive” with 4 = “No Effect.”

*MSPSS*, Multidimensional Scale of Perceived Social Support; *IES-R*, Impact of Events Scale – Revised. *PTGI*, Post Traumatic Growth Inventory; *TCF*, team cohesion factor.
